# Survey of incidence, lifetime prevalence, and treatment of self-reported vulvovaginal candidiasis, United States, 2020

**DOI:** 10.1186/s12905-022-01741-x

**Published:** 2022-05-10

**Authors:** Kaitlin Benedict, Alyson L. Singleton, Brendan R. Jackson, Noelle Angelique M. Molinari

**Affiliations:** Division of Foodborne, Waterborne, and Environmental Diseases, National Center for Emerging and Zoonotic Infectious Diseases, Centers for Disease Control and Prevention, 1600 Clifton Road NE, Mailstop H24-9, Atlanta, GA 30329 USA

**Keywords:** Candidiasis, vulvovaginal, Incidence, Prevalence, Antifungal Agents, United States

## Abstract

**Background:**

Vulvovaginal candidiasis (VVC) is a common gynecologic problem in the United States but estimates of its true incidence and prevalence are lacking. We estimated self-reported incidence and lifetime prevalence of healthcare provider-diagnosed VVC and recurrent VVC (RVVC), assessed treatment types, and evaluated demographic and health-related risk factors associated with VVC.

**Methods:**

An online survey sent to 4548 U.S. adults; data were weighted to be representative of the population. We conducted descriptive and bivariate analyses to examine demographic characteristics and health related factors associated with having VVC in the past year, lifetime prevalence of VVC, and over-the-counter (OTC) and prescription antifungal treatment use. We conducted multivariate analyses to assess features associated with 1) having VVC in the past year, 2) number of VVC episodes in the past year, and 3) lifetime prevalence of VVC.

**Results:**

Among the subset of 1869 women respondents, 98 (5.2%) had VVC in the past year; of those, 5 (4.7%) had RVVC. Total, 991 (53%) women reported healthcare provider-diagnosed VVC in their lifetime. Overall, 72% of women with VVC in the past year reported prescription antifungal treatment use, 40% reported OTC antifungal treatment use, and 16% reported both. In multivariate analyses, odds of having VVC in the past year were highest for women with less than a high school education (aOR = 6.30, CI: 1.84–21.65), with a child/children under 18 years old (aOR = 3.14, CI: 1.58–6.25), with diabetes (aOR = 2.93, CI: 1.32–6.47), who were part of a couple (aOR = 2.86, CI: 1.42–5.78), and with more visits to a healthcare provider for any reason (aOR = 2.72, CI: 1.84–4.01). Similar factors were associated with increasing number of VVC episodes in the past year and with lifetime prevalence of VVC.

**Conclusion:**

VVC remains a common infection in the United States. Our analysis supports known clinical risk factors for VVC and suggests that antifungal treatment use is high, underscoring the need to ensure appropriate diagnosis and treatment.

## Background

Vaginal symptoms such as itching, pain, and discharge are among the most common reasons for visits to gynecologic healthcare providers, and vaginitis is associated with substantial morbidity that negatively affects patients’ physical and mental health [1]. Approximately one-third of vaginitis cases are caused by *Candida* [2]. Known risk factors for vulvovaginal candidiasis (VVC) include antibiotic use, hormonal contraceptive use, pregnancy, and diabetes [3]. Recurrent vulvovaginal candidiasis (RVVC) is usually defined as ≥ 4 VVC episodes per year and is difficult to treat [4].

VVC is frequently self-diagnosed or empirically diagnosed by healthcare providers, and no public health surveillance for these infections exists in the United States, making incidence and prevalence calculations challenging [4]. Widely cited statistics that 75% of women will experience VVC in their lifetime and 5–10% will experience RVVC are not well supported by epidemiologic evidence [5]. A recent systematic literature review estimated that RVVC affects nearly 6 million women in the United States each year, but more robust primary data are needed to better understand the current burden of these infections and inform prevention strategies [6]. Previous studies have suggested racial disparities in VVC rates, but the topic has not been recently studied or comprehensively assessed in the context of other potentially related demographic features or medical risk factors [3, 7].

We analyzed data from U.S. adult women to estimate self-reported incidence and lifetime prevalence of healthcare provider diagnosed VVC and RVVC, assess treatment types, and evaluate demographic and health-related risk factors associated with VVC.

## Methods

### Survey methods

Porter Novelli, a public relations firm, conducted an online survey in fall 2020. The survey covered various health topics, including four questions about healthcare provider diagnosed VVC frequency and treatment (Table 1). Survey participants were randomly recruited from Ipsos’ nationally representative KnowledgePanel® by mail using address-based probability sampling to reach panel members regardless of whether they had Internet access, and participants were provided with a laptop or tablet computer and Internet access if needed. The survey was sent to 4548 people ages 18 or older, 3625 (79.7%) of whom completed it. Survey weights were designed to reflect U.S. adult population demographic proportions, rather than totals, of gender, age, household income, race/ethnicity, household size, education, census region, and metropolitan status using the March 2019 Consumer Population Survey benchmark proportions.

**Table 1:** Vulvovaginal candidiasis questions and response options—Porter Novelli Fall ConsumerStyles Survey, United States, 2020

Question	Response options
Among female respondents:	Yes, no, don’t know/don’t remember
In the past 12 months, has a healthcare provider diagnosed you with a vaginal yeast infection?	
Among respondents who answered “yes” to question 1:	Numeric
In the past 12 months, how many times has a healthcare provider diagnosed you with a vaginal yeast infection?	
Among respondents who answered “yes” to question 1:	Select all that apply: over-the counter antifungal treatment, alternative/non-medicinal treatment, prescription antifungal treatment, none of the above
Which of the following did you use for your vaginal yeast infection?	
Among female respondents:	Never, 1 time, 2–3 times, 4–5 times, 6–10 times, more than 10 times, don’t know/don’t remember
In your lifetime, how many times has a healthcare provider diagnosed you with a vaginal yeast infection?	

### Data analysis

All analyses were weighted and restricted to female respondents. We conducted descriptive and bivariate analyses to examine demographic characteristics and health related factors associated with having VVC in the past year, lifetime prevalence of VVC, over-the-counter (OTC) antifungal treatment use, and prescription antifungal treatment use. RVVC was defined as ≥ 4 episodes of VVC in the past year.

We conducted multivariate analyses to assess demographic characteristics and health related factors associated with (1) having VVC in the past year, (2) number of VVC episodes in the past year, and (3) lifetime prevalence of VVC. Having VVC in the past year was estimated via weighted logistic regression, yielding estimates of adjusted odds ratios (aORs); number of VVC episodes was estimated via weighted negative binomial regression to address overdispersion, yielding estimates of adjusted incidence rate ratios (aIRRs); and lifetime prevalence of VVC was estimated via weighted ordered logistic regression, yielding proportional aORs. We modeled increasing number of VVC episodes rather than incidence of RVVC due to small numbers of respondents who reported RVVC. Explanatory variables with likelihood ratio test p values smaller than 0.30 were retained in the regressions. Standard errors were bootstrapped with 250 replications for robust estimation of 95% confidence intervals (CIs).

Analyses were conducted using SAS (version 9.4; SAS Institute). CDC licensed this data from Porter Novelli. While Porter Novelli and Ipsos are not subject to CDC Institutional Board review, they do adhere to all professional standards and codes of conduct set forth by the ESOMAR Code of Conduct (https://esomar.org/code-and-guidelines/icc-esomar-code) and the Insights Association (https://www.insightsassociation.org/issues-policies/insights-association-code-standards-and-ethics-market-research-and-data-analytics-0). Respondents were informed that their answers were being used for market research and they may refuse to answer any question at any time. No personal identifiers were included in the data file provided to CDC.

## Results

### Descriptive and bivariate analyses

Among 1869 women, 98 (5.2%) reported that a healthcare provider had diagnosed them with VVC in the past year (Table 2); of these, 5 (4.7%) had RVVC. Total, 991 (53%) women said that a healthcare provider had ever diagnosed them with VVC in their lifetime; among women ≥ 60 years, this proportion was 59%. The largest proportion (34%) of women with VVC in the past year were 30–44 years old; most were white (74%) and non-Hispanic (84%) (Table 2). Over half (54%) of women with VVC in the past year had some college education or more, most (79%) were part of a couple, 48% had a child/children under 18 years old, and 22% had diabetes.

**Table 2:** Demographic and health characteristics of all female respondents, females who reported having vulvovaginal candidiasis (VVC) in the past year, and antifungal treatment—Porter Novelli Fall ConsumerStyles Survey, United States, 2020

	All females		VVC in the past year			Over-the-counter antifungal treatment			Prescription antifungal treatment		
	Weighted n	%	Weighted n	%	*p* value*	Weighted n	%	*p* value* †	Weighted n	%	*p* value* †
Total	1869		98	5.2		39	40.2		71	72.2	
Age group in years					0.44			0.36			0.04
18–29	349	18.7	16	16.7		9	22.0		13	18.9	
30–44	469	25.1	33	34.1		15	39.0		24	34.2	
45–59	472	25.3	23	23.2		10	24.8		10	14.3	
≥ 60	579	31.0	26	26.0		6	14.2		23	32.7	
Race**					0.64			0.36			0.34
White	1429	76.5	72	73.9		32	80.5		50	69.9	
Non-white or multiracial	440	23.5	26	26.1		8	19.5		21	30.1	
Ethnicity					0.88			0.56			0.86
Non-Hispanic	1599	85.6	83	84.9		35	88.5		60	85.4	
Hispanic	270	14.4	15	15.1		5	11.5		10	14.6	
Education					0.01			0.33			0.80
Less than high school	171	9.1	19	19.3		9	21.8		15	20.9	
High school	590	31.6	26	26.7		8	19.5		18	25.5	
Some college	525	28.1	34	34.4		11	29.0		26	36.6	
Bachelor's degree or higher	583	31.2	19	19.5		12	29.8		12	17.0	
Couple status					0.01			0.003			0.05
Part of a couple	1187	63.5	78	79.3		37	94.6		52	74.1	
Single	682	36.5	20	20.7		2	5.4		18	25.9	
Have child(ren) < 18 years old					0.001			0.19			0.003
Yes	536	28.7	47	47.7		23	58.6		26	37.2	
No	1333	71.3	51	52.3		16	41.4		44	62.8	
Perceived health					0.25			0.98			0.67
Good	1608	86.1	80	81.4		32	81.2		57	80.1	
Not good	261	13.9	18	18.6		7	18.8		14	19.9	
Median number of healthcare provider visits in the past year (IQR)	1.0	0–2	2.0	0–3	< 0.001	2.0	1–2	0.55	2.0	1–3	0.35
Diabetes	183	9.8	22	22.3	0.001	11	28.0	0.39	18	26.1	0.23
Median household size (IQR)	3.0	2–4	3.0	2–4	0.38	3	2–5	0.22	2.0	1–4	0.03
Median income in thousands (IQR)	67.5	37.5–112.5	67.5	32.5 – 112.5	0.20	80.0	45– 112.5	0.13	55.0	22.5 –112.5	0.002
Metropolitan statistical area category					0.73			0.73			0.18
Non-metropolitan	256	13.7	12	11.9		6	14.1		11	14.9	
Metropolitan	1613	86.3	86	88.1		34	85.9		60	85.1	
Census region					0.28			0.77			0.85
Northeast	328	17.6	13	13.4		8	18.9		9	12.9	
Midwest	415	22.2	15	15.1		5	13.6		10	13.6	
South	710	38.0	42	43.2		17	43.2		30	42.1	
West	416	22.2	28	28.3		10	24.3		22	31.3	

IQR = Interquartile range

^*^p value of the c^2^ test for association †for treatment versus no treatment, among women with VVC

^**^Survey participants could check all that apply from: American Indian or Alaskan Native, Asian, Black or African American, Native Hawaiian or Other Pacific Islander, White, Other, Don’t know

Overall, 72% of women with VVC in the past year reported prescription antifungal treatment use, 40% reported OTC antifungal treatment use (Table 2), 16% reported both, 5% reported using an alternative or nonmedicinal treatment, and 2% reported no treatment (Table 3). More women reported using both OTC and prescription antifungal treatments with increasing number of VVC episodes in the past year (Table 3). All women with RVVC reported using both OTC and prescription antifungal treatment.

**Table 3:** Vulvovaginal candidiasis (VVC) treatment, by number of VVC episodes in the past year—Porter Novelli Fall ConsumerStyles Survey, United States, 2020

	VVC (n = 93)						RVVC (n = 5)	
	1 episode (n = 61)		2 episodes (n = 23)		3 episodes (n = 9)		4–5 episodes (n = 5)	
	Weighted n	%	Weighted n	%	Weighted n	%	Weighted n	%
Over-the-counter antifungal only	22	36.5	0	0.0	1	0.0	0	0.0
Prescription antifungal only	34	55.2	18	76.4	3	76.4	0	0.0
Over-the-counter and Prescription antifungal	3	5.6	5	23.6	2	23.6	5	100.0
Alternative/nonmedicinal treatment	0	0.0	0	0.0	1	0.0	0	0.0
No treatment	2	2.7	0	0.0	1	0.0	0	0.0

OTC and prescription antifungal treatment use did not differ substantially by age, race, ethnicity, educational status, metropolitan vs. non-metropolitan status, or Census region. In bivariate analyses, women were more likely to use OTC antifungal treatment if they were part of a couple (38.1% vs. 2.2%, *p* = 0.0028, OR = 7.97, CI: 1.55–40.93) and were more likely to use prescription antifungal treatment if they had no child under 18 years old (44.4% vs. 26.3%, *p* = 0.0029, OR = 0.198, CI: 0.062–0.632) or had lower median household income ($55,000 vs. $93,000, *p* = 0.0018, OR = 0.83, CI: 0.74–0.93).

### Multivariate analyses

The odds of having VVC in the past year were highest for women with less than a high school education (aOR = 6.30, CI: 1.84–21.65), followed by those with some college (aOR = 2.62, CI: 1.21, 5.67) compared with those with a bachelor’s degree or higher (Table 4). The odds of having VVC in the past year were also higher for women with a child/children under 18 years old (aOR = 3.14, CI: 1.58–6.25), women with diabetes (aOR = 2.93, CI: 1.32–6.47), women who were part of a couple (aOR = 2.86, CI: 1.42–5.78), and women with more healthcare provider visits for any reason (aOR = 2.72, CI: 1.84–4.01). Similar factors were associated with increasing number of VVC episodes in the past year: having a child/children under 18 years old (aIRR = 2.21, CI: 1.04–4.69), having diabetes (aIRR = 2.76, CI: 1.25, 6.10), being part of a couple (aIRR = 3.30, CI: 1.61–6.77), and increasing number of healthcare provider visits for any reason (aIRR = 3.06, CI: 2.13–4.41). An increase in income from $60,000–$74,999 to $75,000–$84,999 was associated with a 10% (CI: 3–16%) decline in number of VVC episodes in the past year. We did not observe significant differences in having VVC or with number of VVC episodes with regards to age, race, ethnicity, or metropolitan vs. non-metropolitan status. Odds of having VVC and expected number of VVC episodes were slightly elevated in the South Census region, though these effects were not strong.

**Table 4:** Demographic and health characteristics associated with having vulvovaginal candidiasis (VVC) in the past year, number of VVC episodes in the past year, and VVC lifetime prevalence—Porter Novelli Fall ConsumerStyles Survey, United States, 2020 (n = 1773)*

	VVC in the past year				Number of VVC episodes in the past year				VVC lifetime prevalence			
	aOR	95% CI		*P* value	aIRR	95% CI		*P* value	aOR	95% CI		*P* value
Age in years	0.99	0.97	1.01	0.409	0.90	0.75	1.07	0.240	1.12	1.07	1.17	< 0.0001
Race												
White	Ref	Ref	Ref	0.482	Ref	Ref	Ref	0.396	Ref	Ref	Ref	0.104
Non-white or multiracial	1.27	0.65	2.50		0.75	0.38	1.45		1.25	0.95	1.64	
Ethnicity												
Non-Hispanic	Ref	Ref	Ref	0.479	Ref	Ref	Ref		Ref	Ref	Ref	0.375
Hispanic	0.68	0.23	1.99						0.84	0.58	1.23	
Education												
Less than high school	6.30	1.84	21.65	0.019	1.59	0.80	3.15	0.113	0.88	0.50	1.55	0.149
High school	2.19	0.93	5.15		2.80	0.98	7.97		0.98	0.74	1.29	
Some college	2.62	1.21	5.67		2.14	1.06	4.31		1.29	1.00	1.66	
Bachelor's degree or Higher	Ref	Ref	Ref		Ref	Ref	Ref		Ref	Ref	Ref	
Couple status												
Part of a couple	2.86	1.42	5.78	0.004	3.30	1.61	6.77	< 0.0001	1.22	0.95	1.58	0.116
Single	Ref	Ref	Ref		Ref	Ref	Ref		Ref	Ref	Ref	
Have child(ren) < 18 years old												
Yes	3.14	1.58	6.25	0.001	2.21	1.04	4.69	0.040	1.63	1.24	2.15	0.000
No	Ref	Ref	Ref		Ref	Ref	Ref		Ref	Ref	Ref	
Perceived health	0.76	0.54	1.05	0.095	0.75	0.54	1.04	0.072	1.05	0.93	1.19	0.438
Number of healthcare provider visits in the past year	2.72	1.84	4.01	< 0.0001	3.06	2.13	4.41	< 0.0001	1.40	1.22	1.61	< 0.0001
Diabetes	2.93	1.32	6.47	0.008	2.76	1.25	6.10	0.004	1.59	1.06	2.37	0.025
Household size	0.89	0.71	1.10	0.279	0.91	0.74	1.12	0.344	0.93	0.84	1.03	0.187
Income	0.96	0.90	1.03	0.254	0.90	0.84	0.97	0.004	1.02	0.99	1.05	0.213
Census region												
Northeast	Ref	Ref	Ref	0.100	Ref	Ref	Ref	0.103	Ref	Ref	Ref	0.191
Midwest	0.68	0.21	2.196		0.67	0.33	1.35		1.23	0.87	1.73	
South	1.66	0.58	4.73		1.71	0.89	3.29		1.41	1.02	1.94	
West	1.97	0.67	5.78		0.93	0.41	2.10		1.18	0.84	1.66	
Model fit statistics												
AIC	679.06				786.85				5147.97			
− 2logL	645.06				750.85				5103.97			
Max rescaled R squared	0.19								0.12			
Degrees of freedom	16	1757			16	1756			17	1756		

^*^Explanatory variables for multivariate analyses included: age in years as a 2nd degree polynomial, race, ethnicity, education, status as part of a couple, having a child/children under 18 years old, perceived health scaled from 1 (excellent) to 5 (poor), number of healthcare provider visits in the past year, diabetes, household size, income (< $5000–$14,999 in increments of $2499; $15,000–$34,999 in increments of $4,999; $40,000–$59,999 in increments of $9999; $60,000–$99,999 in increments of $14,999; $100,000–$199,999 in increments of $24,999; $200,000– $249,999; and > $250,000), Metropolitan Statistical Area category, and Census region of residence

Lifetime prevalence of VVC increased with age (aOR = 1.12, CI: 1.07–1.17); a 10-year increase in age above the median age was associated with a 120% increase in the odds of moving to the next highest ordered category. Controlling for other factors, higher odds of having VVC in the lifetime were associated with: having a child/children under 18 years old (aOR = 1.63, CI: 1.24–2.15), diabetes, (aOR = 1.59, CI: 1.06–2.37), and an increase from 1 to 2 healthcare provider visits for any reason in the past year, which increased the odds by 40% (CI: 22–61%).

## Discussion

Results from this survey provide an updated estimate of self-reported VVC incidence and lifetime prevalence among adult women in the US, confirming that it remains a common infection. Estimating the true public health burden of VVC remains challenging given that it is likely underdiagnosed.

In this study, the annual incidence of self-reported healthcare provider diagnosed VVC (5.2%) and RVVC among those with VVC (4.7%) suggests that ~ 6.8 million women experience VVC and ~ 325,000 experience RVVC in the United States each year, based on an estimated 2019 population of 130,851,717 women age 18 and older 8. Although the number of survey respondents reporting RVVC in the past year was small, these projections show that the number of VVC and RVVC cases may be substantial, in accordance with a previous study that showed a large healthcare burden of nearly 1.4 million outpatient visits and $374 million in direct medical costs related to VVC each year [9]. Our estimate of self-reported healthcare provider diagnosed VVC are remarkably similar to a previous incidence estimate of 5.6% from a telephone survey of U.S. women conducted during 1991–1996 [7, 10]. Similarly, our estimate of RVVC incidence is consistent with an estimate of 5% in 2013 among an internet survey of U.S. and European women with VVC [7, 11].

Robust population-based studies about the lifetime prevalence of VVC and RVVC are scarce. Previously, the most comprehensive study, upon which the recent estimates of the global burden of VVC are based [6], found that the lifetime prevalence of self-reported healthcare provider diagnosed VVC in the United States and 5 European countries ranged from 29 to 49% by country, lower than our estimate of 53% [12]. Also, nearly a quarter of women in the previous study who reported having VVC also reported having RVVC at some point in their lifetime, and the lifetime prevalence of RVVC among all women was 9%; [12] however, our survey did not ask about lifetime prevalence of RVVC.

### Strengths and limitations

The overall representativeness of the survey is a strength of our study. Our study’s primary limitation is the self-reported nature of the data, which are subject to misclassification and recall challenges. We chose to ask about provider diagnosed, rather than self-diagnosed VVC, consistent with most of the previous incidence and lifetime prevalence estimates [7, 12], Given the nonspecific nature of VVC signs and symptoms, both overdiagnosis and underdiagnosis by healthcare providers are potential concerns. In addition, some women with VVC likely do not seek medical care because of self-diagnosis and OTC treatment [7], However, the accuracy of self-diagnosis of VVC also tends to be poor, even among women with previous VVC episodes [7, 13]. Another limitation is that the survey did not capture experiences of non-English speakers. Estimating the incidence and lifetime prevalence of VVC is further complicated by many factors likely influencing health status and healthcare seeking behaviors.

Our multivariate analyses of features associated with having VVC in the past year, number of VVC episodes in the past year, and lifetime prevalence of VVC appear to support known clinical risk factors for VVC, namely, diabetes and hormonal contraceptive use (as approximated by partnered status and having children under 18 years old) [3, 14]. Increasing number of healthcare provider visits could indicate poorer overall health status, higher antibiotic use, higher willingness to seek medical care, or easier access to medical care. Similarly, education and income might also be associated with overall health status. Our results do not point to racial/ethnic disparities in VVC after controlling for other potentially related factors. In contrast, a 1995 survey of self-reported provider diagnosed VVC found a higher prevalence of VVC and other vaginal symptoms among Black women compared with white women, though Black women were more likely to seek healthcare [10, 15]. Although we were not able to specifically study RVVC due to small sample sizes, demographic and health-related features associated with increasing number of VVC episodes were similar to those associated with having VVC in the past year, suggesting that explanations unaccounted for in this analysis (for example, genetic susceptibility) may predispose women to developing RVVC [3].

Although geography was not significantly associated with VVC or number of VVC episodes in the multivariate analyses, our results point towards a potentially larger burden of VVC in the Southern U.S. compared with other regions. This phenomenon has not been well described but was also evident in a previous study of nationwide outpatient visit data [7]. Higher rates of outpatient fluconazole prescriptions also support a disproportionate burden of VVC in the South [16].Whether this disparity reflects differences in VVC diagnostic practices or susceptibility to VVC is unknown but deserves further study. Rates of bacterial sexually transmitted infections and antibiotic use are also higher in the South, which could lead to increased risk for developing VVC [17, 18].

Most women (72%) with VVC in this survey reported prescription antifungal use, and a moderate proportion (40%) reported OTC antifungal use, with increasing proportions of women using both prescription and OTC antifungals as number of episodes increased, perhaps reflecting VVC infections not responsive to initial treatment, misdiagnosis of other vaginal infections as VVC, or both. Short-course intravaginal OTC antifungals are often sufficient to treat uncomplicated VVC, whereas prescription antifungals are preferred for severe or recurrent infections or immunocompromised women [19, 20]. Given the self-reported nature of these data, we were not able to determine appropriateness of treatment; however, previous studies show that antifungal drug misuse is common with VVC, both with OTC antifungals to treat self-diagnosed VVC and with inappropriate antifungal prescribing by healthcare providers [2, 21]. In addition to the possibility for prolonged symptoms, inappropriate use of these medications raises broader concerns for the development of antifungal resistance, an emerging clinical and public health problem. This is especially important because the majority of outpatient antifungal prescriptions are fluconazole and likely used to treat VVC [16]. We were not able to identify factors associated with OTC antifungal use or prescription antifungal use with multivariate analyses due to limited sample size. However, we observed bivariate associations with marital status, children in the home, household size, and income, suggesting that socioeconomic status and access to care, among many other factors, likely influence treatment-seeking decisions. Continuing to understand the epidemiology of VVC will be critical in light of the recent approval of ibrexafungerp, the first drug in a new antifungal class in more than 20 years.

## Conclusions

Altogether, results from this nationally representative survey of adult women help contribute to an increased understanding of the estimated incidence and lifetime prevalence of VVC in the United States. Because this common infection can be associated with substantial morbidity and economic costs, increased attention to accurate diagnosis and appropriate treatment is warranted. Our results further support known clinical risk factors for VVC, underscoring the continued importance of targeting health messaging to women at higher risk and to healthcare providers regarding VVC prevention, recognition, and treatment.

## Data Availability

CDC licensed the Fall 2020 ConsumerStyles dataset used for the current study from Porter Novelli. The data are closed to the public but are available from Porter Novelli: https://www.porternovelli.com/.
